# Bimodal behaviour of charge carriers in graphene induced by electric double layer

**DOI:** 10.1038/srep30731

**Published:** 2016-07-28

**Authors:** Sing-Jyun Tsai, Ruey-Jen Yang

**Affiliations:** 1 Department of Engineering science, National Cheng Kung University, Tainan 701, Taiwan

## Abstract

A theoretical investigation is performed into the electronic properties of graphene in the presence of liquid as a function of the contact area ratio. It is shown that the electric double layer (EDL) formed at the interface of the graphene and the liquid causes an overlap of the conduction bands and valance bands and increases the density of state (DOS) at the Fermi energy (*E*_*F*_). In other words, a greater number of charge carriers are induced for transport and the graphene changes from a semiconductor to a semimetal. In addition, it is shown that the dependence of the DOS at *E*_*F*_ on the contact area ratio has a bimodal distribution which responses to the experimental observation, a pinnacle curve. The maximum number of induced carriers is expected to occur at contact area ratios of 40% and 60%. In general, the present results indicate that modulating the EDL provides an effective means of tuning the electronic properties of graphene in the presence of liquid.

The generation of electric energy by a liquid flowing over a carbon nanotube (CNT) was first reported by Kral and Shapiro in 2001^1^. According to their theoretical model, energy generation occurs as the result of momentum transfer induced by the flowing liquid, which produces a phonon wind and drags the free charge carriers in the nanotube. The electric generation effect has been confirmed by several experimental studies^2^,^3^,^4^,^5^. However, the generated energy typically has a value of less than 10 mV. Moreover, ion transport in CNTs is complicated by various factors^6^,^7^, including the entrance effect, interface effect, and nanotube type (e.g., metal/semiconductor/multilayer, and so on). Consequently, the problem of identifying more suitable materials for the generation of electricity via fluid flows has attracted significant interest in recent years. Of the various new materials which have emerged over the past decade or so, graphene (a carbon-related material) has attracted particular attention due to its superior transport properties^8^,^9^,^10^,^11^,^12^,^13^ and high sensitivity to external fields^14^,^15^,^16^,^17^. Thus, the potential for utilizing graphene as a medium for energy conversion has received extensive attention in the recent literature.

Various experimental studies have confirmed the feasibility for generating electric energy by flowing liquid over a graphene surface^18^,^19^,^20^,^21^,^22^,^23^,^24^. It has been reported that the induced voltage is proportional to the number of droplets passing over the surface^23^. Furthermore, for the case where the graphene is plunged into a NaCl aqueous solution, the variation of the induced voltage with the exposed area has the form of a pinnacle curve^24^. However, when the graphene is totally immersed in the solution, and therefore has no liquid-gas boundary, the voltage reduces sharply to zero. In a previous investigation into water flow over a graphene surface, it was found that while an induced voltage was initially detected, it gradually reduced over time^20^. Notably, such a phenomenon is not observed in water flows over a CNT and cannot be explained by any of the mechanisms previously proposed for nanotubes^1^,^2^,^3^,^4^,^5^. Thus, it has been suggested that the reduction in the induced voltage in graphene systems may be a result of such factors as a net drift velocity of the adsorbed ions (ion drag), phonon drag, or a moving boundary of the electric double layer^18^,^19^,^20^,^21^,^22^,^23^,^24^. However, these factors do not easily explain the observed relation between the induced voltage and the contact area. As a result, the exact origin of the induced voltage in graphene is unclear and requires further investigation, both theoretically and experimentally.

For many substances, an EDL is formed at the interface of the substrate surface when placed in contact with a liquid (e.g., water, aqueous solution, and so on)^25^,^26^,^27^. The EDL consists of two layers. The first layer results from the adsorbed charges (ions) on the substance surface (via chemical interactions), while the second layer is formed by the counter ions attracted from the solution by the surface charges (via Coulomb force). The charge distribution of the EDL is complicated. However, it has been shown that the electric potential can be described as an exponential function decay from the interface to the bulk solution^27^. In general, the potential may vary from 10 mV to as much as 50 mV within a distance of several hundreds of nanometers from the surface. As a result, the EDL plays a key role in many microfluidic transport phenomena^28^, including electroosmosis, electrophoresis, and the streaming potential. Many microfluidic devices have been proposed for performing energy conversion by manipulating the EDL^29^,^30^,^31^,^32^,^33^. For example, Krupenkin and Taylor^29^ used the reverse electro-wetting effect induced by a moving array of liquid droplets to generate electric power with an intensity proportional to the number of moving droplets. Moon *et al*.^31^ investigated the electrical power generated by liquid flows over an ITO surface, and showed that the voltage exhibited a quadratic dependence on the contact area.

As with many other materials, graphene forms an EDL when brought into contact with liquids^34^,^35^,^36^,^37^ and provides the ability to generate energy by modulating the EDL^18^,^19^,^20^,^21^,^22^,^23^,^24^. In general, charged particles of liquid or gas are easily adsorbed on graphene and form a layer of surface charges^38^,^39^,^40^. The adsorption energy depends mainly on the ions species of solution in contact with the graphene surface and can be evaluated using density functional theory^41^ and experiment^42^. For example, the adsorption energy of the hydrated Na^+^ ion within a NaCl aqueous solution is around 0.1 eV^43^. These surface adsorbates induce a doping effect to modulate the *π*-electronic structures of the graphene^44^,^45^, and therefore also impacts the transport properties (e.g., the conductance and the mobility). Moreover, the transport properties of graphene are highly sensitive to changes in the external field^8^, and hence the induced voltage is expected to show a similar sensitivity. However, the effect of liquid on the electronic properties of graphene still requires fundamental theoretical viewpoints. Moreover, the relation between the induced voltage and the exposed area of the graphene surface to liquid remains unclear. Thus, to fully understand the behavior of the charge carriers in graphene-liquid systems, it is imperative to further investigate the effect of the EDL on the *π*-electronic structures of graphene.

Accordingly, this study performs a theoretical investigation into the electronic properties of graphene in the presence of liquid for various values of the contact area ratio. To more easily manipulate the contact area ratio, and to increase the total exposure area so as to enhance the generated power, the investigation considers the system shown in Fig. 1(a), in which a monolayer graphene is placed in contact with a microfludic chip composed of a periodic array of microchannels (arranged along the armchair direction) filled with liquid. The period length and channel width are set as L and w, respectively. Thus, the ratio of the exposure area in a fixed period can be tuned by the contact ratio w/L. The EDL formed at the interface between the graphene monolayer and the liquid prompts the formation of an effective electric field (Fig. 1(c)) in a direction perpendicular to the graphene surface. To investigate the resulting behavior of the charge carriers in the graphene, a tight-binding model^17^ is used to calculate the low-energy dispersions and density of states (DOS) near the Fermi level. The dependence of the energy band and density of state on the contact area ratio is systematically explored. The theoretical results provide a useful insight into the optimal value of w/L, i.e., the value of w/L at which the maximum number of charge carriers are prospectively induced for transport.

## Results

**Figure 1:**
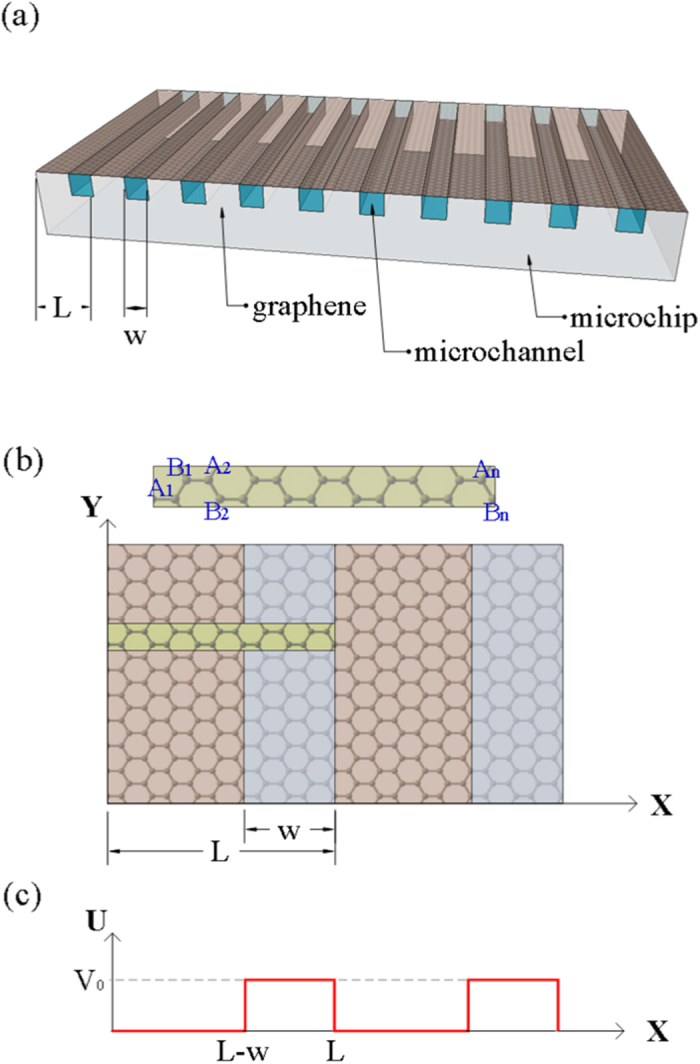
(**a**) Schematic illustration of graphene monolayer in contact with periodic array of microchannels filled with liquid. (**b**) Enlarged primitive unit cell (green rectangular zone) in effective-modulated electric field caused by EDL. Note that blue (pink) region shows graphene in presence (absence) of EDL. (**c**) The effective-modulated electric potential profile caused by EDL.

**Figure 2:**
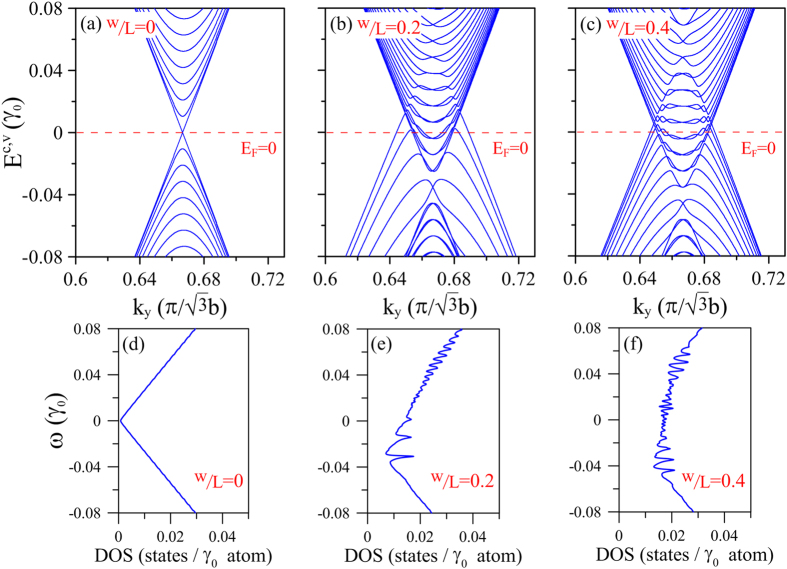
*k*_*y*_-dependent low-energy bands near Dirac point: (**a**) in absence of liquid (w/L = 0) with enlarged unit cell for *R*_*E*_ = 300, and in presence of liquid with *R*_*E*_ = 300 and *V*_0_ = 0.1 *γ*_0_ (~0.25 eV) at: (**b**) w/L = 0.2 and (**c**) w/L = 0.4. (**d**–**f**) corresponding DOS for cases (**a**~**c**).

Due to the period of the effective electric potential, the primitive unit cell of the combined graphene-liquid system is larger than that of pristine graphene. To compare the results for the energy bands in the absence and presence of liquid, respectively, the unit cell of pristine graphene in this paper is rearranged as an enlarged one like other cases under electric field (Fig. 1(b)). Due to the zone folding method, the energy bands in the hexagonal Brillouin zone (BZ) are folded into a reduced rectangular BZ. Notably, the folded energy bands retain all the characteristics of the original band structure. Moreover, the corresponding DOS also remains unchanged. For period lengths greater than 80 nm, the low-energy dispersion depends strongly on *k*_*y*_, but varies only weakly with *k*_*x*_^17^. Thus, the present study focuses specifically on the *k*_*y*_-dependent energy spectrum. For pristine graphene, the conduction bands and valance bands are symmetric and meet at the Fermi level. In other words, the graphene acts as a zero-gap semiconductor (Fig. 2(a)). The lowest conduction band and highest valance band have a linear dispersion relation around the Dirac points. Moreover, the low-frequency DOS is linearly related to the frequency *ω* and has a value close to zero at the Fermi level (Fig. 2(d)). Thus, the charge carriers in the graphene are sensitive to the external field and can be transported rapidly, while no free carriers exist at the Fermi energy.

When the graphene is exposed to the liquid in the microchannels with a contact ratio of w/L = 0.2 (Fig. 2(b)), the conduction bands and valence bands overlap at *E*_*F*_, and some Fermi-momentum states are induced. Moreover, the low-energy bands exhibit oscillations, which result in new band-edge states. The changes in the energy bands will directly reflect on the density of states. For example, the DOS at the Fermi level is raised to a finite value due to the produced Fermi-momentum states, and a large number of prominent peaks are generated as a result of the induced band-edge states (Fig. 2(e)). Moreover, the number of induced electron states exceeds the number of hole states. The increased DOS(*E*_*F*_) implies that charge carriers are generated by the EDL and further contribute to the voltage induced by the external field. For a larger contact area ratio of w/L = 0.4, the energy dispersions become more oscillatory, and hence more band-edge states are produced (Fig. 2(c)). Furthermore, more minor peaks are induced in the DOS, and the value of the DOS at the Fermi level is increased (Fig. 2(f)). Consequently, the number of charge carriers generated for transport is also increased, i.e., the graphene becomes more metallic. In general, the results presented in Fig. 2 confirm the feasibility for tuning the carrier density of graphene through an appropriate manipulation of the EDL.

**Figure 3:**
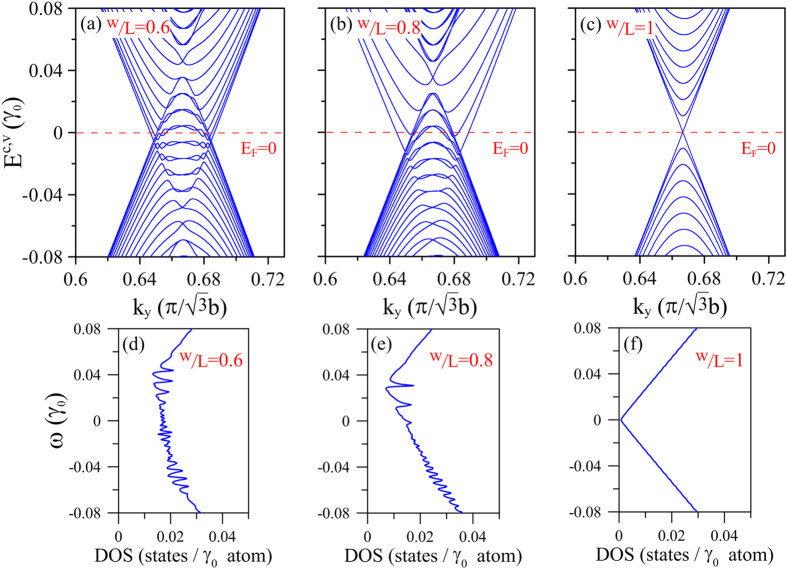
*k*_*y*_-dependent low-energy bands near Dirac point in presence of liquid with *R*_*E*_ = 300 and *V*_0_ = 0.1 *γ*_0_ (~0.25 eV) at: (**a**) w/L = 0.6, (**b**) w/L = 0.8, and (**c**) w/L = 1. (**d**–**f**) corresponding DOS for cases (**a**~**c**).

Due to the symmetry of the graphene lattice, the band structure returns to symmetric about the Fermi level at a contact ratio of w/L = 0.5 (not shown). In other words, the number of induced electron states is equal to the number of generated hole states. For contact ratios away from w/L = 0.5, the energy dispersions exhibit an inversion symmetry distribution. For example, given a contact ratio of w/L = 0.6, the energy bands are inverted with respect to those of w/L = 0.4 about the Fermi level (Figs 2(c) and 3(a)). This is coming from that the unit cells of w/L = 0.4 and w/L = 0.6 under effective electric potential exhibit an inversion symmetry as well. As a result, the corresponding DOS is also inverted (Figs 2(f) and 3(d)) and has the same value at the Fermi level. However, the number of induced hole states (induced electron states) at w/L = 0.6 is greater (less) than that at w/L = 0.4. As w/L further increases to 0.8 (Fig. 3(b)), the energy dispersions become the inversion of those at w/L = 0.2 through the Fermi level. Furthermore, the valence bands are more condensed than the conduction bands. In addition, the number of induced hole states is greater than the number of electron states (Fig. 3(e)). That is to say the majority-carrier type can be tuned by contact area ratio. As the w/L < 0.5, the majority carriers are electrons, while they are holes for w/L > 0.5. For w/L = 1, the energy bands are restored to those of pristine graphene, and the DOS distribution is identical to that in the absence of liquid. In other words, when the graphene is completely covered with solution, the DOS at *E*_*F*_ reverses to zero. As a result, no free carriers exist for transport, and hence the induced voltage reduces to zero^24^.

**Figure 4:**
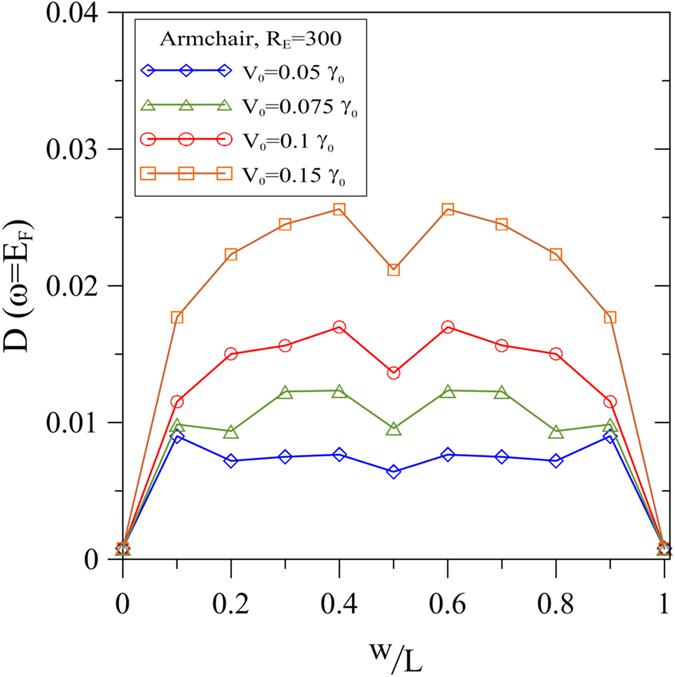
DOS at Fermi level as function of contact ratio w/L given various values of *V*_0_ and constant *R*_*E*_ = 300.

To further explore the dependence of the induced charge carriers on the contact ratio, Fig. 4 plots the variation of the DOS at the Fermi level with the contact ratio w/L as a function of the field strength (*V*_0_). It is noted that the DOS curves are symmetric about w/L = 0.5 for all values of the field strength due to the inversion symmetry of the energy spectrum. For *V*_0_ = 0.05 *γ*_0_, the DOS increases rapidly to a maximum value at w/L = 0.1, and then decreases slightly and oscillates as the contact ratio approaches w/L = 0.5. For contact ratios greater than w/L = 0.9, the DOS falls rapidly to a value close to zero. The maximum value of the DOS(*ω* = 0) indicates the presence of the maximum number of induced charge carriers at the corresponding contact ratio. For a field strength of *V*_0_ = 0.075 *γ*_0_, the maximum DOS(*ω* = 0) occurs at a contact ratio of w/L = 0.3~0.4. Furthermore, for a field strength of *V*_0_ = 0.1 *γ*_0_, the dependence of DOS(*ω* = 0) on w/L shows a bimodal distribution with maximum values located at w/L = 0.4 and w/L = 0.6, respectively. For the highest considered field strength of *V*_0_ = 0.15 *γ*_0_, the bimodal curve has an even more pronounced characteristic, with strong peaks once again located at w/L = 0.4 and w/L = 0.6, respectively. In general, the results show that under a higher field strength, the induced charge carriers are more abundant and more densely concentrated at specific contact area ratios. In addition, owing to the asymmetry between electron and hole conductivities^46^,^47^,^48^ (hole conductivity is higher than electron one), it is predicted that the induced voltage exhibits an asymmetrically bimodal dependence on contact ratio. The asymmetric-bimodal behaviour is consistent with that reported experimentally for graphene immersed in ionic solution^24^. Due to the charge ions, the field strength of ionic liquid is stronger than that of DI water. Therefore, the variation of the induced voltage with the exposure area has the form of a pinnacle curve, and is thus consistent with the DOS(*ω* = 0) tendency. Moreover, no induced voltage is detected when the graphene is totally isolated and plunged into water. Furthermore, Lee *et al*. found that the detected voltage along the flow direction is higher than that along the normal direction to the flow^20^,^21^. According to a previous study^17^,^49^, the group velocity perpendicular to the modulation direction is reduced. As a result, the liquid distributed on graphene surface along a particular direction provides an effective modulation of the electric field, and therefore provides the means to tune the energy dispersion of graphene. On the other hand, multilayer graphene (such as bi-layer and tri-layer) owns distinct band structure from that of monolayer one owing to different crystallographic stacking sequences and interlayer interactions^50^,^51^,^52^. Therefore, multilayer systems are expected to display different dependence on graphene-liquid contact ratio.

## Discussion

This study has utilized a tight-binding model to investigate the electronic properties of graphene in the presence of fluid for various values of the graphene-fluid contact area ratio. Results have been presented for both the low-energy bands and the density of states near the Fermi level. It has been shown that pristine graphene is a zero-gap semiconductor, and has no charge carriers at the Fermi level. In addition, the results have shown that the electric double layer formed at the interface between the graphene surface and the fluid results in an effective electric potential. The electric field causes the conduction bands and valence bands to overlap, and consequently increases the DOS at *E*_*F*_ from zero to a finite value. In other words, the EDL induces some free carriers, which contribute to the voltage produced by the external electrical field. As the contact area increases, the DOS(*E*_*F*_) does not increase monotonically, but exhibits a bimodal distribution. In other words, the charge carriers do not increase proportionally with the exposure area, but are enriched initially and then decrease as w/L continues to rise.

For a field strength greater than 0.25 eV, the maximum number of free carriers is induced at contact ratios of 40% and 60%, respectively, and gives rise to corresponding peaks in the induced voltage signal. Notably, the number of induced carriers varies not only with the contact area ratio, but also with the field strength of the EDL, which depends in turn on the type of liquid in contact with the graphene. In addition, the majority-carrier type is tunable by changing contact area ratio. The calculation results obtained in this study are consistent with the experimental observations^20^,^21^,^23^,^24^ and confirm the feasibility of graphene-based energy conversion devices. Moreover, the results show that modulating the EDL provides a flexible and effective approach for tuning the electronic properties of graphene.

## Methods

The graphene monolayer shown in Fig. 1(b) comprises carbon atoms arranged in a honeycomb lattice. The primitive cell consists of two carbon atoms (labeled as A and B, respectively), with each carbon atom owning four outer electrons. Three of these electrons are connected to neighboring carbon atoms via *σ*-bonds^9^, while the other electron (referred to as the *π* electron) is free for transport. As a result, the low-energy electronic properties are governed mainly by the 2*P*_*z*_ orbitals. The eigenstate of the system can be represented using two tight-binding Bloch functions, i.e.,


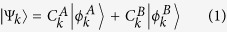


where 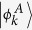 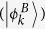 is the superposition of the 2*P*_*z*_ orbitals belonging to periodic A (B) atoms.

For the arrangement shown in Fig. 1(a), the interfaces between the liquid and the graphene form a sequence of EDLs (Fig. 1(b), blue zone), which result in a periodic electric field at the surface of the graphene. The electric field can be described as an effective electric potential modulated by the field strength *V*_0_, period length *L*, and contact ratio w/L (Fig. 1(c)). In previous studies^17^,^49^, the low-energy bands are not sensitive to the modulation direction (armchair or zigzag) of modulated electric potential. Thus, for convenience in defining the primitive unit cell, the period L is designed as 3*bR*_*E*_ along the armchair direction, where *b* = 1.42 Å is the c-c bond length and *R*_*E*_ is a positive-integer parameter used to adjust the period length. In addition, *V*_0_ is set about 0.1–0.4 eV responding to different adsorption potential strength, and scaled by *γ*_0_. Here, *γ*_0_ is the hopping integral between the nearest neighboring atoms A and B, the value (~2.59 eV) can be obtained from the Slonczewski-Weiss-McClure model^53^,^54^. It is generally utilized to set the energy scale for the system. In the present study, the characteristics of the charge carriers in the graphene are investigated by calculating the low-energy bands and DOS near the Fermi level as a function of both the contact area ratio and the electric field strength.

Due to the effective electric potential, the graphene primitive cell is enlarged and contains 4*R*_*E*_ carbon atoms (*A*_1_, *B*_1_, *A*_2_, *B*_2_, …, 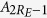, 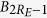, 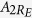 and 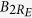) (Fig. 1(b), green zone). The eigenfunction of such a system is composed of 4*R*_*E*_ tight-binding Bloch functions (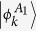, 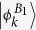, 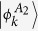, 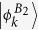, … 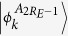, 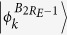, 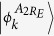 and 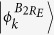), and has the form


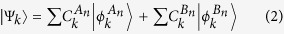


In the above basis, the Hamiltonian is expressed as a 4*R*_*E*_ * 4*R*_*E*_ Hermitian matrix. To enhance the computational efficiency^17^, the basis is rearranged as (*A*_1_, 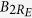, *B*_1_, 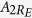, …, 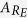,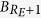, 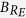 and 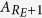), such that the Hamiltonian can be rewritten as a band-like matrix^17^. By adopting this strategy, an enlarge unit cell consisting of approximately 1200 atoms (the corresponding period length ~0.12 *μ*m) can be considered simultaneously. The energy spectrum of the system can be solved by diagonalizing the band-like Hermitian matrix. In addition, the features of the electronic structures impact directly on the DOS, and hence the density of states can be calculated as


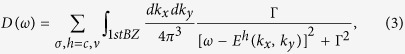


where Γ is the phenomenological broadening parameter owing to various de-excitation mechanisms, e.g., the electron–electron, electron–phonon, electron–impurity scattering. In this study, Γ is treated as a constant (Γ~ 1.3 meV). Note that in investigating the behavior of the charge carriers, the present study focuses specifically on the low-energy bands and the DOS near the Fermi level.

## Additional Information

**How to cite this article**: Tsai, S.-J. and Yang, R.-J. Bimodal behaviour of charge carriers in graphene induced by electric double layer. *Sci. Rep.* **6**, 30731; doi: 10.1038/srep30731 (2016).

## References

[b1] Kral P. & Shapiro M. Nanotube electron drag in flowing liquids. Phys. Rev. Lett. 86, 131–134 (2001). 11136111 10.1103/PhysRevLett.86.131

[b2] Ghosh S., Sood A. K. & Kumar N. Carbon nanotube flow sensors. Science 299, 1042–1044 (2003). 12532025 10.1126/science.1079080

[b3] Ghosh S., Sood A. K., Ramaswamy S. & Kumar N. Flow-induced voltage and current generation in carbon nanotubes. Phys. Rev. B 70, 205423 (2004).

[b4] Liu J. W., Dai L. M. & Baur J. W. Multiwalled carbon nanotubes for flow-induced voltage generation. J. Appl. Phys. 101, 064312 (2007).

[b5] Lee S. H., Kim D., Kim S. & Han C. S. Flow-induced voltage generation in high-purity metallic and semiconducting carbon nanotubes. Appl. Phys. Lett. 99, 104103 (2011).

[b6] Park H. G. & Jung Y. Carbon nanofluidics of rapid water transport for energy applications.Chem. Soc. Rev. 43, 565–576 (2014). 24141359 10.1039/c3cs60253b

[b7] Noy A. . Nanofluidics in carbon nanotubes. Nano. Today 2, 22–29 (2007).

[b8] Geim A. K. & Novoselov K. S. The rise of graphene.Nat. Mater. 6, 183–191 (2007). 17330084 10.1038/nmat1849

[b9] Novoselov K. S. . Electronic properties of graphene. Phys. Status. Solidi. B 244, 4106–4111 (2007).

[b10] Nomura K. & MacDonald A. H. Quantum transport of massless dirac fermions.Phys. Rev. Lett. 98, 076602 (2007). 17359041 10.1103/PhysRevLett.98.076602

[b11] Yazyev O. V. & Louie S. G. Electronic transport in polycrystalline graphene.Nat. Mater. 9, 806–809 (2010). 20729847 10.1038/nmat2830

[b12] Das Sarma S., Adam S., Hwang E. H. & Rossi E. Electronic transport in two-dimensional graphene.Rev. Mod. Phys. 83, 407–470 (2011).

[b13] Wang S. T. . Direct probing of density of states of reduced graphene oxides in a wide voltage range by tunneling junction. Appl. Phys. Lett. 101, 183110 (2012).

[b14] Zhang Y. B., Tan Y. W., Stormer H. L. & Kim P. Experimental observation of the quantum Hall effect and Berry’s phase in graphene. Nature 438, 201–204 (2005). 16281031 10.1038/nature04235

[b15] Lukose V., Shankar R. & Baskaran G. Novel electric field effects on Landau levels in graphene.Phys. Rev. Lett. 98, 116802 (2007). 17501075 10.1103/PhysRevLett.98.116802

[b16] Yan J., Zhang Y. B., Kim P. & Pinczuk A. Electric field effect tuning of electron-phonon coupling in graphene. Phys. Rev. Lett. 98, 166802 (2007). 17501446 10.1103/PhysRevLett.98.166802

[b17] Ho J. H., Chiu Y. H., Tsai S. J. & Lin M. F. Semimetallic graphene in a modulated electric potential.Phys. Rev. B 79, 115427 (2009).

[b18] Dhiman P. . Harvesting Energy from Water Flow over Graphene. Nano. Lett. 11, 3123–3127 (2011). 21749100 10.1021/nl2011559

[b19] Yin J., Zhang Z. H., Li X. M., Zhou J. X. & Guo W. L. Harvesting Energy from Water Flow over Graphene? Nano. Lett. 12, 1736–1741 (2012). 22381077 10.1021/nl300636g

[b20] Lee S. H., Jung Y., Kim S. & Han C. S. Flow-induced voltage generation in non-ionic liquids over monolayer graphene. Appl. Phys. Lett. 102, 063116 (2013). 10.1186/1556-276X-8-487 PMC4225506 24252646

[b21] Lee S. H. . Flow-induced voltage generation over monolayer graphene in the presence of herringbone grooves. Nanoscale Res. Lett. 8, 487 (2013). 24252646 10.1186/1556-276X-8-487 PMC4225506

[b22] Uesugi E., Goto H., Eguchi R., Fujiwara A. & Kubozono Y. Electric double-layer capacitance between an ionic liquid and few-layer graphene.Sci. Rep. 3, 1595 (2013). 23549208 10.1038/srep01595 PMC3615339

[b23] Yin J. . Generating electricity by moving a droplet of ionic liquid along graphene. Nat. Nanotechnol. 9, 378–383 (2014). 24705513 10.1038/nnano.2014.56

[b24] Yin J. . Waving potential in graphene. Nat. Commun. 5, 3582 (2014). 24800734 10.1038/ncomms4582

[b25] Parsons R. Electrical Double-Layer - Recent Experimental and Theoretical Developments. Chem. Rev. 90, 813–826 (1990).

[b26] Attard P. Recent advances in the electric double layer in colloid science. Curr. Opin. Colloid. In. 6, 366–371 (2001).

[b27] Kirby B. J. & Hasselbrink E. F. Zeta potential of microfluidic substrates: 1. Theory, experimental techniques, and effects on separations.Electrophoresis 25, 187–202 (2004). 14743473 10.1002/elps.200305754

[b28] Kirby B. Micro- and nanoscale fluid mechanics: transport in microfluidic devices (Cambridge Univ. Press, 2010).

[b29] Krupenkin T. & Taylor J. A. Reverse electrowetting as a new approach to high-power energy harvesting.Nat. Commun. 2, 448 (2011). 21863015 10.1038/ncomms1454 PMC3265368

[b30] Chang C. C. & Yang R. J. Electrokinetic energy conversion efficiency in ion-selective nanopores. Appl. Phys. Lett. 99, 083102 (2011).

[b31] Moon J. K., Jeong J., Lee D. & Pak H. K. Electrical power generation by mechanically modulating electrical double layers.Nat. Commun. 4, 1487 (2013). 23403587 10.1038/ncomms2485

[b32] Miljkovic N., Preston D. J., Enright R. & Wang E. N. Electrostatic charging of jumping droplets.Nat. Commun. 4, 2517 (2013). 24071721 10.1038/ncomms3517

[b33] Miljkovic N., Preston D. J., Enright R. & Wang E. N. Jumping-droplet electrostatic energy harvesting.Appl. Phys. Lett. 105, 013111 (2014).

[b34] Newaz A. K. M., Markov D. A., Prasai D. & Bolotin K. I. Graphene Transistor as a Probe for Streaming Potential. Nano. Lett. 12, 2931–2935 (2012). 22568874 10.1021/nl300603v PMC4433749

[b35] Park J. S. . Wetting and Evaporative Aggregation of Nanofluid Droplets on CVD-Synthesized Hydrophobic Graphene Surfaces. Langmuir 30, 8268–8275 (2014). 24559308 10.1021/la404854z

[b36] Liu J., Ye Z. C., Zhang L., Fang X. M. & Zhang Z. G. A combined numerical and experimental study on graphene/ionic liquid nanofluid based direct absorption solar collector. Sol. Energ. Mat. Sol. C 136, 177–186 (2015).

[b37] Miller J. R., Outlaw R. A. & Holloway B. C. Graphene electric double layer capacitor with ultra-high-power performance. Electrochim. Acta. 56, 10443–10449 (2011).

[b38] Schedin F. . Detection of individual gas molecules adsorbed on graphene. Nat. Mater. 6, 652–655 (2007). 17660825 10.1038/nmat1967

[b39] Casolo S., Lovvik O. M., Martinazzo R. & Tantardini G. F. Understanding adsorption of hydrogen atoms on graphene. J. Chem. Phys. 130, 054704 (2009). 19206986 10.1063/1.3072333

[b40] Cole D. J., Ang P. K. & Loh K. P. Ion Adsorption at the Graphene/Electrolyte Interface.J. Phys. Chem. Lett. 2, 1799–1803 (2011).

[b41] Leenaerts O., Partoens B. & Peeters F. M. Water on graphene: Hydrophobicity and dipole moment using density functional theory.Phys. Rev. B 79, 235440 (2009).

[b42] Li H., Lu T., Pan L., Zhang Y. & Sun Z. Electrosorption behavior of graphene in NaCl solutions.J. Mater. Chem. 19, 6773–6779 (2009).

[b43] Jiang D.-E. & Chen Z. Graphene Chemistry: Theoretical Perspectives (Wiley, 2013).

[b44] Liu H., Liu Y. & Zhua D. Chemical doping of graphene.J. Mater. Chem. 21, 3335–3345 (2011).

[b45] Pinto H. & Markevich A. Electronic and electrochemical doping of graphene by surface adsorbates, Beilstein J. Nanotechnol. 5, 1842–1848 (2014). 25383296 10.3762/bjnano.5.195 PMC4222436

[b46] Hwang E. H., Adam S. & Das Sarma S. Carrier Transport in Two-Dimensional Graphene Layers.Phys. Rev. Lett. 98, 186806 (2007). 17501596 10.1103/PhysRevLett.98.186806

[b47] Huard B., Stander N., Sulpizio J. A. & Goldhaber-Gordon D. Evidence of the role of contacts on the observed electron-hole asymmetry in graphene. Phys. Rev. B 78, 121402 (2008).

[b48] Farmer D. B. . Chemical Doping and Electron-Hole Conduction Asymmetry in Graphene Devices. Nano Lett. 9, 388–392 (2009). 19102701 10.1021/nl803214a

[b49] Park C.-H., Yang Li, Son Y.-W., Cohen M. L. & Louie, Steven G. New generation of massless Dirac fermions in graphene under external periodic potentials. Phys. Rev. Lett. 101, 126804 (2008). 18851401 10.1103/PhysRevLett.101.126804

[b50] Oostinga J. B., Heersche H. B., Liu X., Morpurgo A. F. & Vandersypen L. M. K. Gate-induced insulating state in bilayer graphene devices.Nat. Mater. 7, 151–157 (2007). 18059274 10.1038/nmat2082

[b51] Craciuna M. F., Russob S., Yamamotoc M. & Taruchac S. Tuneable electronic properties in graphene. Nanotoday 6, 42–60 (2011).

[b52] Lui C. H., Li Z., Mak K. F., Cappelluti E. & Heinz T. F. Observation of an electrically tunable band gap in trilayer graphene. Nat. Phys. 7, 944–947 (2011).

[b53] Wallace P. R. The band theory of graphite. Phys. Rev. 71, 622 (1947).

[b54] Charlier J. C., Gonze X. & Michenaud J. P. First-principles study of the electronic properties of graphite. Phys. Rev. B 43, 4579–4589 (1991). 10.1103/physrevb.43.4579 9997825

